# Orofacial pain/headache interlaced to insomnia, sleep apnea and periodic limb movement during sleep/restless leg syndrome: a critical and comprehensive review with insights into social determinants

**DOI:** 10.22514/jofph.2025.023

**Published:** 2025-06-12

**Authors:** Alberto Herrero Babiloni, Cibele Dal Fabbro, Flavia P. Kapos, Helena Hachul, Gilles J. Lavigne

**Affiliations:** ^1^Stomatology Department, Center for Advanced Research in Sleep Medicine, Sacre Coeur Hospital, CIUSSS du Nord d’Ile de Montreal, Montreal, QC H4J 1C5, Canada; ^2^Instituto do Sono, 04020-060 Sao Paulo, SP, Brazil; ^3^Faculty of Dental Medicine, Université de Montréal, Montreal, QC H3T 1J4, Canada; ^4^Department of Orthopaedic Surgery & Duke Clinical Research Institute, Duke University School of Medicine, Durham, NC 27710, USA; ^5^Departamento de Psicobiologia, Universidade Federal de São Paulo, 04023-062 Sao Paulo, SP, Brazil; ^6^Division of Experimental Medicine and Faculty of Dental Medicine and Oral Health Sciences, McGill University, Montreal, QC H4A 3J1, Canada

**Keywords:** COMISA, Management, Orexin, Pain, Sleep, Social determinants

## Abstract

This critical review explores the intricate interaction between orofacial pain and headache disorders and three prevalent sleep disorders: insomnia, obstructive sleep apnea (OSA) and periodic limb movements (PLMs) during sleep, while integrating the role of social determinants of health. Orofacial pain conditions, including temporomandibular disorders (TMD), headache, and burning mouth syndrome, frequently co-occur with sleep disturbances, creating a bidirectional cycle where poor sleep exacerbates pain and *vice versa*. The mechanisms underlying this relationship involve disrupted restorative sleep, neuroinflammation, heightened arousal and impaired descending pain modulation. Importantly, social factors such as socioeconomic status, healthcare access, education level, and social support influence the prevalence, severity, and management of these comorbidities, contributing to significant disparities in outcomes. We present recent advances in the phenotyping and endotyping of individuals with sleep-pain comorbidities, which aim to identify subgroups with shared characteristics to guide personalized interventions, emphasizing the need for interdisciplinary approaches that bridge dentistry, sleep medicine and public health to address the multifactorial nature of these conditions. Practical considerations for clinicians managing these patients are discussed, including screening tools, treatment modalities and the impact of social context. Future research directions prioritize the integration of measures of social factors into study designs, advancing personalized medicine and employing innovative technologies to better understand genotypes and phenotypes involved in pain perception and sleep characteristics, and manage the interplay between sleep and orofacial pain. The goal of addressing the interaction between sleep and pain is to improve health equity and optimize outcomes for individuals affected by these interrelated conditions.

## 1. Introduction

Pain is defined as “an unpleasant sensory and emotional experience associated with actual or potential tissue damage or described in terms of such damage”. When this pain persists or recurs for longer than 3 months, it is named chronic pain. Several chronic pain conditions have been associated with poor sleep quality. Individuals with chronic pain conditions such as widespread pain (*e.g.*, fibromyalgia), chronic low back pain or chronic overlapping pain conditions (COPCs), frequently report disturbed sleep and related fatigue complaints [1, 2]. Sleep disturbances, such as poor sleep quality, unstable sleep continuity due to frequent sleep awakening, and unrefreshed sleep perception, are also associated with orofacial pains and headache disorders, such as temporomandibular disorders (TMD), tension-type headache, migraine, cluster headache, burning mouth syndrome (BMS), odontogenic pain such as third molar pain, and, to a certain extent, trigeminal neuralgia [3, 4, 5, 6, 7, 8, 9].

During the last few years, there have been advances in the understanding of the interaction between sleep and pain disorders, considering both as joint interlaced problems rather than as separate comorbidities. More evidence has been gathered about the directionality of this association, which seems to be stronger in the direction of sleep disorders causing pain problems [10]. For instance, it has been recently proposed that treating sleep disorders should be considered a first line of treatment in chronic pain patients [11, 12], and multiple authors have advocated for adequate screening of sleep disturbances among this population, as well as comprehensive assessment and management if needed to prevent chronic pain development. Recent research has also focused on the identification of subgroups of patients that share common features in order to identify casual pathways (*i.e.*, phenotyping), and the recognition of putative mechanistic links underlying different phenotypes (*i.e.*, endotyping) [13]. In any case, different sleep disturbances and sleep disorders may contribute differently to different pain conditions or symptoms, and this becomes particularly relevant in the field of orofacial pain and headache disorders, where different pain and sleep disorders are interlaced. These elements point out the intricate interplay of biological, environmental and psychosocial factors that shape individual responses and outcomes. Genotypic variability as well, influences susceptibility to specific conditions. For instance, *catechol-O-methyltransferase* (*COMT*) gene polymorphisms have been identified as risk factors for TMD susceptibility [14], while phenotypic and endotypic expressions modulate the clinical presentation and progression of TMD and other chronic pain disorders [13, 15]. Furthermore, individual threshold levels, shaped by both intrinsic and extrinsic factors, dictate the degree to which stimuli are perceived and processed [16, 17], adding another dimension to this multifaceted relationship. These overlapping and interdependent layers highlight the challenges inherent in disentangling causative pathways and identifying modifiable risk factors, not only in sleep and pain [15, 18, 19, 20] but also in many other health conditions [21, 22, 23, 24]. This complexity not only obscures our ability to generalize findings across populations but also emphasizes the need for robust methodologies and personalized approaches in research and clinical practice [25]. A nuanced understanding of these associations will require integrating multi-dimensional data from genetic, molecular and clinical studies while considering the socio-environmental context.

In that way, the recognition of the contribution of social determinants of health, or the social aspects of the biopsychosocial model of pain, have emerged as pivotal aspects in understanding the complex interplay between interlaced health conditions, such as sleep and chronic pain disorders. Factors such as socioeconomic status, education level, access to healthcare, occupational stress and social support can significantly influence both sleep quality and pain perception, and inequities in these social factors may exacerbate the burden of both sleep disturbances and chronic pain, particularly in marginalized populations. For instance, individuals experiencing financial insecurity or social isolation may face heightened risks of disrupted sleep and chronic pain due to increased stress and reduced access to timely and adequate care [26, 27, 28, 29]. Moreover, cultural and societal norms may shape how individuals perceive and report pain and sleep disturbances, further complicating diagnosis and treatment. Incorporating these dimensions into research and clinical care not only broadens our understanding of the sleep-pain nexus, but also underscores the need for holistic, patient-centered approaches that address biopsychosocial dimensions of health.

The purpose of this review is to describe the interaction between sleep and orofacial pain/headache in relation to three sleep disorders: insomnia, sleep breathing disorders/obstructive sleep apnea (SBD/OSA), and periodic limb movements (PLMs) during sleep, the latter often associated with restless legs syndrome (RLS) during wakefulness. These sleep disorders frequently co-occur, exacerbating pain in vulnerable or at-risk individuals through independent or synergistic effects on the comorbid status. Beyond the biological and psychological mechanisms, this review also aims to consider the influence of social determinants of health on pain and sleep, which may impact the development and severity of these conditions. Moreover, we aimed to provide a clinical perspective, describing the role of dentists, physicians, and other health professionals in managing these multifactorial overlaps and emphasizing the importance of interdisciplinary approaches that address not only clinical symptoms but also the broader psychological and social contexts surrounding these disorders.

## 2. Methods

The review process of this critical review involved a search of peer-reviewed articles, book chapters and relevant guidelines published since inception (with no time filter) but with a focus on publications of the last 5 years. This search was supplemented by key foundational and review studies where necessary, including their references. Databases including PubMed, Google Scholar, and Web of Science were searched using combinations of terms such as “sleep disorders”, “nap”, “orofacial pain”, “temporomandibular disorders”, “trigeminal neuralgia”, “burning mouth syndrome”, “idiopathic facial pain”, “headache”, “insomnia”, “obstructive sleep apnea”, “periodic limb movements”, “restless legs syndrome” and “social determinants”. Articles were selected based on their relevance to the outlined topics and their contribution to understanding the biological, psychological and social dimensions of the interaction between these disorders. This type of review was selected to provide a broader narrative synthesis and to address the interdisciplinary and evolving nature of the topics addressed, to describe and structure the information with a clinical orientation.

## 3. Sleep and pain interactions

The connection between sleep and pain has been emphasised in many studies. A theoretical working model, proposed to challenge the impact of such interaction, suggested that bidirectional circular effects between sleep and pain may be considered equipotent until otherwise supported by evidence [2, 3]. In other words, that model suggested that a day with pain will disrupt sleep, and a poor night of sleep will similarly contribute to more pain on the next day. Behind that thinking was the putative cumulative effect of such a process, which disrupts sleep continuity, inducing non-restorative sleep perception, and hence, pain.

Nowadays, evidence suggests that a day of pain may not alter sleep as much as it was initially thought. Indeed, a study of individuals with pain and insomnia with difficulty to initiate or maintain sleep reported that the effect of wake-time pain on sleep was not predominant. Instead, pre-sleep arousal level (potentially enhanced independently of wake-time pain) explained the changes in self-reported sleep quality and, a poor night of sleep predicted next day pain [30]. The association between pre-sleep arousal and poor sleep with more pain on the next day has been reproduced consistently, accumulating strong evidence [31, 32]. Additionally, in this population, the benefit of good sleep on pain has been documented not to last more than the length of the first half day [30]. The short duration of the benefit of good sleep on pain has also been replicated in a general chronic low back pain population divided according to their sleep quality (*i.e.*, poor and good sleep) [33]. In a general population study from Sao Paulo, Brazil, using polysomnography (PSG), it was shown that women with nighttime pain had a decreased quality of life, worsening mood and fatigue [34]. A study of menopausal women with pain and insomnia added precision to our understanding of the sleep and pain interaction; the cyclical effect between pain and sleep appears not to be the same across age and sex. In an actigraphy study, postmenopausal women with high levels of musculoskeletal pain had more sleep duration (*i.e.*, for each 1-unit increase in pain intensity at bedtime, sleep duration increased by an average of 6.7 minutes.), perhaps due to the excess burden of daytime fatigue and pain. But, in addition, sleep duration directly predicted pain intensity the following morning on waking, with a 1-unit increase in pain intensity, for every 6.9 minutes more of sleep [35]. Actigraphy, or activity sensors that provide an estimate of sleep duration, is a valid proxy of sleep activity, but it is less accurate than sleep PSG for counting the number of awakenings and sleep latency in different sleep disorders [36, 37, 38]. The extrapolation of such important findings needs caution since many other unaccounted factors, such as culture, hormonal levels and therapies could have an impact.

To better understand the pain and sleep interaction, a few concepts need to be revisited. Sleep is not anaesthesia or coma; sleep is a state with partial disconnection from the external environment. A sleeping individual remains in direct connection with their own internal physiological milieu to recover from wake time fatigue and activity, *i.e.*, to promote restorative functions. Sleep is also a period to consolidate the memory process of newly learned information or emotional experiences. A sleeping individual does not lose their capacity to react to danger and pain [39, 40, 41]. On the contrary, a sleeping brain keeps the capacity to filter and react to sound, temperature, smell and to identify any incoming menace for body integrity. To recover from wake time fatigue and to restore functions, sleep requires a state of partial isolation from the external environment [3].

The perception of a potentially novel signal may generate arousal from unconscious sleep to a conscious fight or flight awake type of reaction. Sleep arousal is defined as a 3–15-second rise in heart rate and sympathetic cardiorespiratory dominance, with an increase in muscle tone, and a physiological readiness to react. Sleep arousals are repeated 8–15 times per hour of sleep and are age dependent. They are brief and a sleeping individual is unaware of their occurrence. If too frequent or too long, they can disrupt sleep continuity, and this may contribute to a return to consciousness, ranging from partial to full. Studies have demonstrated that the level of reactivity to sound, such as an alarm clock, is sleep-stage dependent (*i.e.*, more responsiveness in light sleep stages N1 and N2, less responsiveness in deep sleep stage N3, and variable in rapid eye movement (REM) sleep), while reactivity to experimental pain is similar across sleep stages [39, 42].

A physiological sleep process, named cyclic alternating pattern (CAP), contributes to the balance of restorative sleep maintenance *vs.* filtering threats to trigger arousal and awakening. Without sleep continuity, the restorative sleep function is perturbed. CAP—called “*phase d’activation transitoire*” (transitory activation phase) by the French doctor who first described it in 1973 (Dr. A Muzet, from Strasbourg, France [43])—is a cyclic mechanism for filtering internal (*e.g.*, body temperature or position, oxygen level, bladder pressure) and external (*e.g.*, room temperature, noise) sensory cues every 20–40 seconds during sleep. CAP is part of an inherited survival mechanism that prepares the body to fight or flee in the presence of a threat or to resume sleep if risk is not perceived. That natural physiological sleep activity is dominant in non-REM sleep, and it is divided into 3 subgroups. The quiet phase is named A1, with a dominance of slow-wave brain activity contributing to restorative function. The A2 phase is a transition from sleep maintenance toward arousal and the A3 phase is marked by arousal and putative reactivity [44, 45]. Individuals with chronic widespread pain/fibromyalgia seem to present a rise of such autonomic activation, associated with more periodic limb movements, and intense breathing events. These patterns are consistent with increased arousal CAP A3, reported to be 50% more prevalent in individuals with fibromyalgia [46, 47]. Further replication of such observations is required before intervention trials of psychological or medication approaches can be performed to establish a stronger cause and effect relationship.

Sleep duration is one of many dimensions of sleep that seem to be altered in individuals with chronic pain (CP). Sleeping less than 6 hours and more than 9 hours have been shown to be associated with greater pain on the next day [48]. Based on a Mendelian randomization study, derived from an European ancestry sample genome-wide association study (GWAS) and single nucleotide polymorphisms (SNP), short sleep duration (less than 6 hours) was described to have a causal relationship with TMD-related pain (modest Odds Ratio (OR): 1.6, 95% Confidence Interval (CI): 1.06–2.41) [49]. This method failed, however, to support such relationship with sleep duration, insomnia, chronotype, snoring and sleep apnea. In a sample of 419 individuals with TMD, where 84% were female, the myalgia-related pain was associated with a statistically significant OR of around 3 for short sleep (*i.e.*, less than 6 hours of sleep) and around 5 for long sleep duration (*i.e.*, over 9 hours) (95% could not be extracted with enough precision from the available figure) [50].

In a comparative sleep laboratory study, our group observed that individuals with chronic widespread pain/fibromyalgia reported shorter sleep and lower sleep efficiency [51]. Indeed, sleep was about 60 minutes shorter, with one less non-REM to REM ultradian sleep cycle; ideally, a person needs 4 to 5 cycles. Sleep efficiency (*i.e.*, the ratio of time sleeping over the time in bed, as a percentage) was also 10% lower in women (*i.e.*, mean and standard error of mean: 83.5 ± 1.6); normal sleep efficiency values are 90–92% and over. Using quantitative analyses of slow-wave sleep, it was also found that women with those pain conditions presented lower power of deep sleep brain wave activity than did men with chronic widespread pain and matched pain-free control women [51].

Although sleep duration is a simple measure, it is only useful when interpreted in light of other sleep measurements such as sleep efficiency, number of sleep stage shifts or other factors such as body movements, sleep arousal, oxygen desaturation, air flow interruption and oro-motor activity. To assess sleep more comprehensively, sleep PSG recording data is needed, including brain activity by electroencephalography (ECG), heart rate by electrocardiography, muscle activity by electromyography (EMG), breathing by air flow and abdominal/rib cage activity and oxygen saturation by oximetry. The interpretation of PSG data must take into consideration factors such as age, sex, body mass index, quality of life and pain, mood/depression and catastrophizing, to increase the accuracy of clinical characterization and recommendations for individuals with diagnosis of OSA and PLMs. For insomnia PSG is only needed if OSA or PLMs or other conditions are concomitant.

Experimental reduction of sleep duration (*i.e.*, sleep deprivation), is also thought to influence pain. A reduction of 4 hours from the usual 8 hours of sleep has been shown to trigger, within 3–4 nights of short sleep, mood and well-being changes in otherwise healthy university subjects, associated with more generalised pain reports and backache [52]. The same group of authors has also shown that healthy subjects took more than 2 days to recover from 3 consecutive weeks of sleep deprivation for 5 nights followed by 2 nights of normal sleep, compared to a group sleeping 8 hours per night. Furthermore, participants reported more spontaneous pain and some individuals seemed more vulnerable than others to hyperalgesia (at 1 week), with habituation to pain or rise in temporal summation (at 3 weeks) under experimental sensory testing [53]. The loss of sleep seems to have different influences over time. Sleep laboratory deprivation studies have consistently shown that lack of sleep contributes to hyperalgesia and that sleep continuity, *i.e.*, reducing arousal, awakening or periodic motor activity, may play an important role in the sleep restorative process [54, 55, 56, 57]. Furthermore, a mediational model showed that such specific experimental disruption of N3 deep sleep, the so-called restorative sleep, increases cellular inflammation by rising levels of Interleukin-6 (IL-6) and Toll-Like Receptor 4 (TLR-4), thus resulting in pain sensitivity in otherwise pain-free individuals [58]. Neuroinflammation is also a critical outcome in individuals with traumatic brain injury who develop pain and/or sleep issues [59]. Furthermore, under experimental sleep deprivation, many cortical activities seem to be blunted in healthy individuals, or altered, including the sensory cortex and decision-making cortical areas of the insula and striatum [60, 61]. The beneficial activity from the descending pain modulatory system is also disrupted under laboratory sleep deprivation protocols, an effect that seems to be more specific to women [62, 63, 64, 65]. Differences have also been observed between healthy subjects and clinical populations; these groups have a different response to sleep deprivation depending on the method used to assess or modulate pain by activation of the endogenous pain system via conditioned pain modulation (CPM) [66].

Individuals with TMD, who participated in placebo analgesia experiments, presented more emotional distress and maladaptive cognitive characteristics relative to individuals without TMD [67, 68, 69]. These responses were race- and sex-dependent; women experience larger conditioning pain relief effects and higher pain relief expectations, whereas white participants reported greater placebo hypoalgesia compared to African American/Black participants. It is noteworthy that placebo analgesia persists during sleep in healthy volunteers and that pharmacological REM sleep deprivation seems to modulate pain relief expectation by enhancing it [70, 71]. Interestingly, what is said to an individual related to pain seems to be processed during sleep and influences the response to pain during sleep assessed the next morning [40]. Furthermore, individuals with chronic TMD-related pain and concomitant insomnia or lower sleep quality presented a lower magnitude of experimental thermal placebo manipulations with a conditioning paradigm [72]. Thus, sleep conditions seem to matter on such experimental paradigms. Additionally, our group also observed that changes in sleep and pain catastrophizing on electronic diaries about prior night increased the risk of perceiving significant pain exacerbations across the day, specifically from the morning (*i.e.*, 6 AM to 12 PM) to the evening (6 PM to 12 PM), highlighting micro-longitudinal important associations in this interplay [73].

## 4. The role of naps

The role of napping in helping CP individuals to cope with poor sleep quality is not well-known. Some people and cultures are prone to nap, having a genetic polyphasic sleep (like cats or dogs who have many bouts of wake and sleep over 24 hours), while for others this sleep behaviour may be a learned familial habit. Indeed, both factors may be involved. Assessing sleep duration and quality in individuals with CP should consider the duration and frequency of the naps. This needs to be differentiated from sudden and frequent sleep during day time–hypersomnolence—due to the possible presence of sleep apnea and subsequent reduction in sleep quality and efficiency [74]. It may seem counterintuitive to assume that napping can help to manage pain. However, some individuals with heart failure, fibromyalgia and hip pain have reported that the presence of napping habits was associated with more pain, mood disorders and poor sleep quality [75, 76, 77]. A telephone survey of nearly 1500 adults aged 55–85 years in the U.S.A corroborates the association of regular napping with pain, depression, and daytime sleepiness; regular napping was more common among higher age groups, ranging from 10% of participants 55–74 years to 25% of participants 75–84 years [78]. Due to the cross-sectional nature of these studies, worse pain could be the cause of napping, or *vice versa*. For instance, in otherwise healthy persons (mean age ± Standard Error of Mean (SEM): 27 ± 1.6 years), hyperalgesia induced experimentally by forced sleep deprivation was reversed by a 30-minute nap [79]. Furthermore, an experimental study also in healthy subjects (mean age ± Standard Deviation (SD): 24.41 ± 5.29) showed that extension of sleep, beyond what they felt to be sufficient, or a midday nap, increased thermal pain tolerance (cold bath) but had no effect on pain thresholds [80]. Therefore, it appears that napping and pain, in the presence of other comorbidities and ageing, have a complex relationship not yet fully clarified. It remains to be demonstrated whether napping could be a complementary treatment strategy for individuals with CP [81]. If a person with CP feels it is helpful to nap, avoiding naps that are too long or too late in the afternoon could minimise the risk of problems to initiate or maintain sleep, and the development of insomnia. Ideally, naps should not be longer than 30 minutes in individuals with insomnia, but this may not have the same relevance in individuals with other comorbidities (*e.g.*, chronic fatigue, CP, mood disorders and depression) or in older adults [82, 83, 84]. A recent meta-analysis showed that individuals with napping duration of 30 min or longer exhibited a higher risk of all-cause mortality, cardiovascular disease and metabolic disease, whereas those with napping durations less than 30 min had no significant risks [85]. Future research may identify individual phenotypes to guide appropriate nap duration and time of day to better control CP effects [86]. One recent study suggest that nap duration does not seem to influence pain related outcomes as measured with the Fonseca Anamnestic Index among adults with TMD [50].

## 5. Orofacial pain/headache and sleep: recognising and managing sleep comorbidities

Considering the prevalence and overlap between insomnia, obstructive sleep apnea (OSA) and RLS in individuals with CP, recognizing and managing each condition to guide health professionals when addressing TMD in the presence of such comorbidities is essential (see Fig. 1). A systematic review (SR) of 37 studies that examine sleep using PSG or diagnosed sleep disorders reported that, among CP individuals with or without an identified diagnosis or cause, the prevalence of insomnia was estimated to be 72%, while the prevalence of OSA and RLS was estimated in 32% [87]. Specifically, among individuals with TMD, two sleep laboratory studies estimated a prevalence of 28% to 52% of OSA, and 36% of insomnia [88, 89]. RLS, assessed with the Cambridge-Hopkins Restless Leg Syndrome Questionnaire Short Form, was present in 17% of individuals who had sought care for TMD in three university-based clinics in Montreal and Boston [90]. Another study, including 2 consecutive overnight polysomnographic studies of a total of 53 individuals with TMD assessed in an Orofacial pain clinic in Maryland and inpatient sleep research facility at Johns Hopkins, U.S.A, estimated a lower prevalence of RLS (n = 2; 4%); the sleep recording prevalence of PLMs, (*i.e.*, PLM activity on EMG >15/h of sleep), was also low at 4% [89].

**Fig. 1.** Prevalence and management overlap for insomnia, obstructive sleep apnea (OSA) and periodic limb movement during sleep (PLMs). RLS: restless legs syndrome.

### 5.1 Insomnia

Insomnia is associated with recurrent self-reported difficulty to initiate or maintain sleep. Individuals with insomnia report dissatisfaction with the quality or quantity of sleep and may experience distress or impairment in important areas of functioning such as working or personal life, behavioural or emotional status. The maintenance of sleep can be a challenge in the middle of the night with difficulties to return to sleep, and insomnia could also be described as an awakening earlier than desired [91, 92]. The sleep latency criterion for insomnia is often defined as longer than 30 minutes. (*i.e.*, taking longer than 30 minutes to fall asleep after getting into bed can contribute to an insomnia diagnosis). Long napping habits or in late afternoon can delay sleep onset, which is an important advice to give to individuals experiencing difficulties to initiate sleep. A long-term population-based study of working-age adults in Finland reported the prevalence of chronic insomnia symptoms (*i.e.*, occurring often or every day) to be 9–10% [93]. In the same study, the prevalence of occasional-irregular insomnia symptoms (*i.e.*, occurring sometimes, or a few times a week or at least a few times a month) was between 40% to 45% in that population. The prevalence of CP in individuals with insomnia has been reported to be 40% in a large European survey with over 80,000 participants [94],

The causes of insomnia are multiple, including anxiety, lifestyle, drug use and hyperarousal [95]. It tends to run in families and can be a learned behaviour, linked or not to genetic vulnerability. To be considered a chronic disorder, insomnia needs to be present 3 times a week for a period of more than 3 months in a regular or irregular manner. Insomnia can be screened with tools such as the Insomnia Severity Index (ISI) self-reported questionnaire, where a cut-off of 15 points is often used to support a diagnosis. However, the impact of comorbidities needs to be assessed as well [96, 97]. The Diagnostic and Statistical Manual of Mental Disorders (DSM) IV or V diagnostic criteria for insomnia has been used in research to assess the association of insomnia with many conditions, including TMD [89, 98]. Sleep diary recording over a week or more is also useful and is frequently used in combination with actigraphy, despite the above-mentioned limitation in accuracy to assess sleep latency and continuity compared to PSG [30, 31, 36]. Sleep testing, with either full PSG in a sleep laboratory or limited testing at home, is not essential if no comorbidities (*e.g.*, sleep apnea or PLMs) are present nor suspected. An important observation is that not all individuals with long sleep onset delay or early morning wake consider themselves insomniacs. Some may take a long time to fall asleep (more than the 30-minute criteria) and use this time to read or listen to music and enjoy. Some who wake up very early in the morning may be adapted to a bi-phasic sleep pattern: upon awakening, they may take this quiet period *e.g.*, to read the newspaper, feed the pets, and then be able to sleep for another 30–90 minutes. It is then important to assess the impact of sleep duration in relation to perception of sleep quality, well-being, functioning, memory, familial and social life.

Reports of non-restorative sleep can occur in otherwise healthy individuals or may be a cardinal sign of insomnia. It is important to note that the feeling of non-restorative sleep is subjective, whereas insomnia is quantifiable, helping a clinician diagnose a sleep disorder. A recent SR of 20 studies from 12 different countries, from which 12 studies were included in the meta-analysis, summarises that insomnia assessed with ISI is reported by 72.9% of 2578 individuals with CP, and poor sleep quality assessed with the Pittsburgh Sleep Quality Index (PSQI) is reported by 75.3% among 3597 individuals with CP [99]. In all studies, the reported mean age was greater than 40 years-old and 16 studies had more female participants than males. In studies using the PSQI, the chronic pain patients had a mean age of 53 ± 12 years and 74.4% were female, whereas in studies using the ISI, the mean age was 63 ± 12 years and 56.7% were female. Furthermore, in another study, problems with initiating and maintaining sleep were associated with higher probability of chronic widespread pain onset at 5 and 18 years of age at 4 years follow-up among adults in a Swedish population-based study, adjusting for age, sex, occupation type, and number of pain sites at baseline (odds ratio, OR point estimates ranging from 1.6–1.9) [100].

The treatment of insomnia ranges from simple sleep hygiene advice to cognitive-behavioural therapy (CBT), and the use of medication, ideally for a short-term. Medications to promote sleep onset or maintenance include the “Z pills”, such as zolpidem or zopiclone. Some off-label medications (*i.e.*, not officially approved for use for insomnia by drug administration agencies such as in Canadian and U.S.A.) are also commonly prescribed for insomnia. For example, trazodone is a low-cost anti-depressive medication used as a sleep pill to stabilise sleep arousal due to its sedative properties. Pregabalin is an anticonvulsant medication often prescribed to individuals with CP due to its sleep-related effect over the putative analgesic ones. Importantly, pregabalin and gabapentin should not be used in combination with opioids, and possibly benzodiazepines due to a risk of central nervous system respiratory depression; both U.S.A. and Canadian drug administration agencies have posted a warning on that matter [101, 102]. Other less frequently used pharmacological medications include doxepine, melatonin agonists (*e.g.*, ramelteon) or orexin antagonists (*e.g.*, suvorexant, lemborexant, daridorexant) [103]. Interestingly, besides “blocking” arousal in order to facilitate the sleep process, orexin pathways have been associated with pain modulation as well, as orexin neurons project to various brain regions involved in pain processing, including the periaqueductal gray (PAG), locus coeruleus (LC) and dorsal raphe nucleus (DRN) [104, 105, 106]. Animal models also suggest that orexin can modulate the neuroinflammatory processes. In humans, however, data is scarce beyond insomnia, although some anecdotal evidence is available when pain disorders are comorbid. For instance, a study among people with fibromyalgia and comorbid insomnia in a double-blind, crossover design, using suvorexant 20 mg versus placebo for 9 nights showed that besides improving several sleep features, suvorexant reduced next-day pain sensitivity on assessments to a radiant heat stimulus [107]. However, a more recent retrospective study using lemborexant 5 mg at night for insomnia in patients with CP and sleep disturbances reported no significant differences in sleep nor pain between those with or without previous use of sleep medications. There was also no statistically significant improvement in pain before, after 2 weeks, and after 4 weeks from treatment initiation [108]. Therefore, more investigation is needed for this medication, which may be appealing in cases of hyperarousal or hyperexcitability. Ideally, sleep pills should be used for short-term treatment, or during a transition period toward a longer benefit treatment such as CBT for insomnia (CBT-I). The long-term benefit of CBT seems superior for individuals with insomnia including those with CP, and it is preferred by both patients and doctors [95, 109, 110]. Alternative approaches, such as exercise, acupuncture and cannabis, may have some level of benefit but better evidence is needed (*e.g.*, subset of patients for whom these treatments would be safe and effective; specific treatment regimens or methods to individualise interventions) before their use can be widely recommended [111, 112, 113, 114]. Specifically for cannabis-based products, current evidence indicates that the magnitude of benefits for individuals with CP is likely not clinically significant. A SR & meta-analysis, from 39 studies (n = 5100, median of the average age 53 years and 53% female), revealed that the benefit of cannabis related product in improving sleep in chronic pain was rather low [115]. Since harm from the medical use of cannabis or cannabinoids has been documented to be more common than benefits, individuals with CP using or considering these products should have medical guidance to assess contraindications and interactions with other substances, manage risks and negative side effects [116]. At this time, recommending use of cannabis for most sleep disorders (*i.e.*, insomnia, OSA or PLMs) is in serious need of solid investigations assessing benefit, risk and safety [114].

Insomnia and pain are highly prevalent in postmenopausal women. This subpopulation presented higher scores of pain when compared to controls (postmenopausal women without insomnia) [117]. It has been reported that about 26–37% of patients with TMD and other orofacial pains are at higher risk of presenting with primary insomnia, assessed by self-report or PSG [89, 118]. Among individuals seeking treatment for orofacial pain, young and older women seem to have higher prevalence of self-reported insomnia than middle-aged women and men [118]. To assess insomnia and sleep quality in individuals with orofacial pain, currently the best tools to be used are the ISI and the PSQI [119]. The severity of insomnia symptoms and changes in sleep quality (*i.e.*, sleep deterioration), assessed with PSQI, seem to be among the predictors of more pain or new onset of TMD [120, 121]. A PSG-based study, limited to female participants with TMD, reported a lower sleep efficiency and more respiratory effort related arousal (RERA) in comparison to female age-matched controls without TMD [122]. Nevertheless, diagnosis of sleep comorbidities in individual with chronic orofacial pain is important. Poor sleep quality is also reported in individuals with burning mouth syndrome (close to 80%), and pain-related awakening was observed in individuals with trigeminal neuralgia (in about 23%). All these provide reasons to believe that it is complex to disentangle the interaction of insomnia or related sleep complaints in clinical populations with orofacial pain [6, 89, 123, 124].

The management of insomnia in individuals with orofacial pain follows the same principles outlined above for individuals with CP; it needs to be personalised to the most relevant complaint (*e.g.*, initiation or maintenance of sleep), and with regard to other comorbidities such as OSA or sleep bruxism [13, 101, 103]. Importantly, if CBT is used, both insomnia and pain could be addressed in a hybrid mode [125]. In our laboratory, we have tested the use of a single dose of trazodone with PSG recordings in a few female participants with TMD to improve sleep stability and/or pain; however, the trial was interrupted. Although some participants felt benefits, one individual developed an adverse vagal reaction. On that note, it is important to recommend the supervision of a physician for off-label prescription of trazodone, and starting at the lowest dose possible [126]. For a summary of qualitative effectiveness based on expert opinion for pain, sleep, and sleep-pain interaction, please see Fig. 2. Complementary and alternative medicine, and non-invasive brain stimulation may also have their role, but this will heavily depend on factors such as participants beliefs or expectations, past experiences, contraindications, compliance or individual treatment responsiveness.

**Fig. 2.** Management strategies for sleep and pain. Legend: +: low; ++: modest; +++: moderate/strong; −: not sufficient evidence. Rating was performed based on expert opinion as a clinical orientation, not as a guideline. Abbreviations: rTMS: repetitive transcranial magnetic stimulation; tDCS: transcranial direct current stimulation; tACS: transcranial alternative current stimulation. tRNS: transcranial random noise stimulation.

If clinical signs and symptoms of active bruxism are noted among patients seeking care for orofacial pain and insomnia or related complaints (*e.g.*, poor sleep quality, dental and/or periodontal structure damage), oral devices, such as interocclusal splints with full arch coverage and bilaterally balanced contacts, may be considered as the first choice for protecting dentoalveolar structures. However, one should remember that these devices also have their risks, as in some patients there is a minor risk of aggravating sleep apnea with the use of stabilization splints [127, 128]. Medications should be a last resource due to limited evidence of its benefits and the potential for negative effects. Some data support the use of medications for sleep bruxism, but they are off-label and not first-line choices in otherwise non-responsive cases. Those include botulinum toxin and low doses of clonidine or clonazepam. Clonazepam had conflicting results for sleep bruxism depending on the population studied [129, 130].

Studies of individuals with primary headaches frequently report associations with insomnia, with prevalence estimates of up to 50%. Tension-type headache and migraines are the headache diagnoses most commonly studied in relation to sleep disturbances; both seem to share common mechanisms of the interactions between sleep and pain described in the above sections [131, 132, 133]. It remains to be fully elucidated if sleep health components influence pain symptoms equally across different pain conditions, including headache disorders. Whether menopausal status (pre-, peri-, early and late menopause based on questionnaires and hormonal blood measures) influences the occurrence of nocturnal awakening with headache (NAH) in females was previously studied in Sao Paulo, Brazil [134]. It was observed that the prevalence of NAH in perimenopausal women was 13.3%. Perimenopause was associated with a higher risk of having NAH (OR: 13.9; 95% confidence interval: 4.3 to 45.2). It was concluded that menopausal status influences NAH and the women in perimenopause presented a high risk of having this complaint. Interestingly, these perimenopausal women presented more complaints of insomnia as well, according to the *Universidade Federal de Sao Paulo* (UNIFESP) Sleep questionnaire [134].

The differential diagnosis for headaches related to sleep patterns should include cluster headache (occurring frequently in the middle of sleep period) and hypnic headache (occurring at sleep onset); each disorder has its own characteristics and management. Furthermore, the clinician should collect the history of medication use and overuse (*e.g.*, assessment for the “medication overuse headache”), alcohol and drug use, history of traumatic brain injury, and possible concomitant sleep disorders (*e.g.*, OSA, sleep bruxism) [135, 136, 137]. The management armamentarium includes CBT and medications adjusted to the individual predominance of either insomnia or headache [133, 138]. It also appears that the orexin system may be involved in migraine [139], medication overuse headache [140], cluster headache [141] and other primary headaches [142]. Interestingly, newer effective therapies for migraine, such as anti-Calcitonin gene-related peptide (CGRP) monoclonal antibodies (mAbs; *e.g.*, erenumab is a mAb to the CGRP receptor, while eptinezumab, fremanezumab and galcanezumab bind to the CGRP molecule) may also have sleep influences directly by acting on CGRP as a sleep modulator, or indirectly, by reducing sleep-disrupting factors such as anxiety. It has been hypothesized that CGRP can also have an influence in sleep-wake regulation, mainly in arousal and wakefulness, as CGRP is known to influence arousal systems [143, 144]. CGRP is highly expressed in the trigeminovascular system and central nervous system regions involved in wakefulness, such as the hypothalamus and brainstem. Thus, increased CGRP activity may promote wakefulness or disrupt sleep by enhancing arousal pathways, hypothetically. Moreover, the hypothalamus, which regulates circadian rhythms and sleep-wake cycles, receives input from CGRP-expressing neurons. CGRP activity in this region may influence the balance between sleep-promoting and wake-promoting signals. Along those lines, a recent Italian multicentric study showed that besides preventing migraine, anti-CGRP medications also improved sleep quality as measured by the PSQI [145]. More research is needed to identify more consistently effects of these molecules on sleep, and in the headache and sleep interaction.

Another important orofacial pain (OFP) whose relationship with sleep is typically assumed, but often understudied, is odontogenic pain [3]. Toothache from dental origin and sleep are closely associated, as dental pain can significantly disrupt sleep quality and patterns. Toothache, often caused by conditions like dental caries, pulpitis or abscesses, activates pain pathways mediated by the trigeminal nerve, which is highly sensitive and densely connected to brain regions involved in pain perception. This activation can lead to difficulty falling asleep, frequent awakenings and reduced overall sleep efficiency. It seems that conditions like OSA [146], sleep bruxism, oral dryness and gastroesophageal reflux may create or exacerbate tooth pain, and possibly disrupt sleep [147]. However, in the case of 3rd molars pathologies (*e.g.*, pericoronitis and impaction), evidence regarding the pain impact on sleep is not that clear, with many studies not assessing it [8, 9], while others recognized it before or after surgery [148, 149].

### 5.2 Obstructive sleep apnea

Obstructive Sleep Apnea (OSA) is characterised by repetitive cessations of breathing (apnea), and low breathing amplitude (hypopnoea) associated with airway obstruction, defined as a ≥90% reduction in airflow for at least 10 seconds for apnea, and ≥30% reduction in airflow for at least 10 seconds, associated with: a drop in oxygen saturation of ≥3–4%, or an arousal from sleep for hypopnea [74]. A population-based study of adults aged 40–85 years in Switzerland (mean body mass index (BMI) = 25.6 kg/m^2^ standard deviation (SD) = 4.1) reported a prevalence of moderate-to-severe sleep breathing disorders (SBD) (*i.e.*, apnea-hypopnoea index (AHI) ≥15/hour of sleep) of 50% for males and 23% for females [150]. Similar findings were reported for the Australian middle-aged population, 47% for males and 24% for females [151]. In the Swiss population, individuals with moderate-to-severe SBD had higher odds of comorbid hypertension, diabetes, metabolic syndrome and depression (OR ranging from 1.9–2.8) [74, 150]. In the Australian middle-aged population, insomnia was reported by 9.2% of males and 15.8% of females, while RLS was reported by 2.2% of males and 3.7% of females; in total, 42.9% of females presented at least one sleep disorder [151].

TMD is frequently reported to be gender dependent, *i.e.*, dominant in females in a ratio of 2–3/1 for males among clinical populations [152, 153], and it seems to be higher in menopausal women than in post-menopausal counerparts according to some studies [154]. Moreover, TMD-induced pain and menopausal symptoms seem to be correlated, and more strongly late menopausal transitions [155]. Nonetheless, mild to modest sleep breathing disturbances were reported in females with TMD, as the frequency of RERA was significantly higher in comparison to control participants (index of 4.3 *vs.* 2.6 RERA/hour of sleep) [122, 156]. Furthermore, it has been shown that women tend to present most of their apnea-hypopnea in REM sleep with a specific pattern: episodes of short duration with a significant hypoxia when long events were observed [157, 158]; the last being associated with higher risk of hypertension, glucose metabolism issues, memory and mood disorders.

Extrapolating from the BMI distribution in the U.S.A. in 2007–2010, the prevalence of moderate-to-severe SBD has been estimated to be 10% and 17% among men aged 30–49 and 50–70 years, respectively, and 3% to 9% in women of these same age groups [159]. In Brazil, a population-based study of adults aged 20–80 years reported a prevalence of moderate-to-severe OSA of 25% among men, and 10% among women. Older age, male sex and obesity were strongly associated with the presence of OSA [160]. The cause of SBD can be genetic/familial (*i.e.*, sharing obesity or cardiovascular or metabolic syndrome risk), anatomical (*e.g.*, retrognathia, deep and narrow palate, large oropharyngeal pillar or tonsil, and presence of the pterygomandibular tendon) or related to one of 3 physiological phenotypes: low muscle responsiveness or tone, overshoot of breathing following oxygen desaturation and rise in carbon dioxide (*i.e.*, high loop gain), or low arousal threshold contributing to sleep instability [20, 161, 162].

The recognition of OSA is often done by screening using the following tools: Epworth Sleepiness Scale (ESS) to assess daytime/wake-time sleepiness, STOP-BANG (which stands for a composite of Snoring history, Tired during the day, Observed stop of breathing while sleeping, high blood Pressure, BMI >35 kg/m^2^ (or 30 kg/m^2^), Age >50 years, Neck circumference >40 cm and male Gender), the Berlin questionnaire, or other questionnaires for snoring and OSA risk [163], such as the NoSAS (Neck circumference, Obesity, Snoring, Age, Sex) [164], as well as clinical examination to assess obstruction risk (*e.g.*, retrognathia, deep and narrow palate, and large oropharyngeal pillar and tonsil assessed by the Mallampati and Friedman classifications, respectively) [20, 74]. A more comprehensive pre-screening clinical tool has been proposed to include BMI, Freidman tonsil score, neck and waist circumference, age (for women only) and sex, with an algorithm that performed with an accuracy of 80% to detect OSA among adults with suspected OSA (*e.g.*, non-restorative sleep or daytime sleepiness not otherwise explained); those studies involved 9 sleep centres worldwide [165]. Morning headache, or headache upon awakening, is a frequent complaint associated with OSA and should be taken in consideration as well [137].

Nowadays, the diagnosis of OSA is performed by a trained physician including a comprehensive evaluation of sleepiness, fatigue, impact on daily functioning, and scores of Apnea-Hypopnoea Index (AHI), Respiratory Disturbance Index (RDI) and Oxygen Desaturation Index (ODI). In addition, the presence or risk of psychiatric (*e.g.*, depression) or medical conditions (*e.g.*, hypertension, coronary artery disease, atrial fibrillation, congestive heart failure, stroke, diabetes, cognitive dysfunction and risk of metabolic syndrome) should be taken into consideration, as well as obesity or BMI. Conditions thought to be impacted by OSA should prompt especially thorough considerations due to the potential consequences of a missed diagnosis. Furthermore, medications that may induce respiratory problems (*e.g.*, opioids, benzodiazepine, pregabalin) must be assessed too, as they can increase the risk of respiratory depression in vulnerable individuals. In normal individuals, such medications may have benefits in perceived sleep quality in the short-term, but negligible or deleterious effects in long-term in objective and subjective assessments. For a more detailed information of how pain medications can affect sleep and *vice versa* see Herrero Babiloni *et al*. [101] 2021. Home sleep testing can be done with oximetry alone, but this may not capture other sleep-related comorbidities such as arousal, PLMs and central sleep apnea. More accuracy is obtained by recording airflow, rib and abdominal cage movement, oximetry, heart rate and ECG. Sleep laboratory testing is recommended in the presence of mild AHI (see category definition below) and difficulty to confirm SBD, when some of the above-listed medical conditions are not controlled, or in the presence of neurological comorbidities, such as REM behaviour disorders, epilepsy or Alzheimer’s disease. Using both home sleep testing with multi recording variables or full-montage sleep laboratory (*i.e.*, PSG study), the commonly used severity scoring categories for AHI and RDI are the following: AHI of 5–14 for mild (threshold for treatment in the presence of medical or psychiatric disorders), 15–29 for moderate (threshold for treatment in the absence of other disorders) and 30 and over for severe. However, current research is showing that these AHI indices are not stand-alone measures and need to be adjusted by considering RERA and oxygen desaturation, with RDI and ODI indices as well [166].

If OSA is not well diagnosed or managed, the risk of morbidity and mortality may rise significantly. This is an important issue of ongoing debate in health sciences [20, 74, 101, 167]. The first-line treatment is the continuous positive airway pressure device (CPAP), which has to be used for more than 4 hours per night and as many nights as possible to have an optimal benefit. Adherence to facial or nasal masks is not easy for every person. The second-line treatment is the mandibular advancement device (MAD), which acts as a lower jaw and tongue forward retainer to maintain airway patency during sleep. The efficacy of MAD in improving OSA metrics is lower than CPAP when comparing the optimal use of both. However, their effectiveness may be similar since, in many individuals, MAD is used slightly more hours per night and more nights per week [20].

In addition, it is often recommended that people with an OSA lose weight (if BMI is high), exercise more and use a sleep positioning device to correct supine position (if it is dominant) that can exacerbate snoring and OSA [74]. CBT can also be used to improve sleep and life hygiene, and to help coping with OSA and its potentially cumbersome treatment. CBT may have better efficacy if insomnia is a comorbidity to OSA, although results have been inconsistent [168, 169, 170]. Surgeries may be indicated to remove nasal or pharyngeal obstruction. Other major and invasive surgeries, such as maxillary/mandibular advancement, bariatric bypass or lingual nerve stimulation have advantages and limitations. Other alternative or emerging therapies that seem to have additive mild benefits for OSA are oropharyngeal muscle training, positional therapy for changing sleep position, acupuncture and medication to reduce nasal inflammation or rhinitis (*e.g.*, topical corticosteroid, decongestant, antihistaminic). Other medications are sometimes used to increase oropharyngeal muscle tone, such as atomoxetine-oxybutynin [171, 172]. It remains to be fully understood if this medication also influences sleep bruxism [173]. Medical cannabis is not recommended by the America Academy of Sleep Medicine for OSA management although a pharmacological Tetrahydrocannabinol (THC) derivative named dronabinol seems to modestly improve AHI [74, 171, 174, 175, 176, 177, 178, 179, 180, 181, 182, 183, 184, 185]. Again, medical monitoring is highly recommended when a person uses cannabis with various mixtures of CBD-THC, especially in combination with other substances.

It is well-known that the use of opioids can aggravate both OSA and central sleep apnea (CSA), besides disturbing sleep architecture and continuity [186]. A SR of 9 studies (n = 3791, age: 50 ± 12 years-old) investigated the prevalence of sleep breathing disorders (SBD), which included snoring, RERA, OSA and central sleep apnea, in both sleep and pain clinics revealed important clinical facts [187]. In pain clinics, 63% of individuals with CP using opioids had SBD, compared to 10% of individuals with CP not using opioids, and to 75% of individuals without CP—not using opioids. CSA was more prevalent among individuals using opioids seen in sleep clinics (33%) than those seen in pain clinics (20%). Another SR confirmed these findings, suggesting that the overall prevalence of central sleep apnea in patients taking chronic opioids was high (24%) [188]. This study also showed that the most important risk factors for severity of CSA were a morphine equivalent daily dosage of >200 mg, and low or normal body mass index. Moreover, CPAP was often ineffective for treating CSA. Another SR indicated a positive association of TMD with OSA. A case study done in Singapore with 86 adults with TMD and upper airway resistance syndrome (a proxy of RERA with fatigue complaint), suggests that RERA was dominant relative to other sleep measures in individuals with TMD [189]. In a large study done in U.S.A adults, Orofacial Pain: Prospective Evaluation and Risk Assessment (OPPERA), the analysis of OSA and TMD association revealed interesting findings from a cohort and a case-control studies. The odds of OSA were assessed with the following proxy: self-report history of sleep apnea or, 2 or more of the following: loud snoring, daytime sleepiness, witnessed apnea and hypertension. OSA diagnosis or severity was not confirmed by PSG or home testing. From the analyses of the case-control study, chronic TMD cases had higher odds (adjusted OR 3.63, 95% CI: 2.03 to 6.52) of having a high incidence of OSA compared with TMD-free controls, adjusting for study site, demographic characteristics, autonomic parameters, BMI and smoking history [190]. In the OPPERA cohort study, individuals with a high likelihood of OSA were more likely to develop first-onset TMD (adjusted hazard ratio, HR 1.7, 95% CI: 1.14 to 2.62), after the same adjustments as in the case-controls study [190]. In 2 PSG studies of individuals seeking care for OSA, TMD was present in about 50% of adult participants [88, 191]. A large population-based cohort study using data from Taiwan National Health Insurance revealed that adults with suspected (based on diagnostic codes) or probable (based on PSG) sleep apnea were more likely to be subsequently diagnosed with TMD (HR 2.5, 95% CI: 1.7 to 3.7) than age and sex-matched controls without OSA randomly selected from the population database [192]. This retrospective study covered a period of 18 years and included over 100,000 controls compared to about 10,000 OSA subjects. The results indicate a sudden rise in TMD diagnosis between years 13–14 after OSA diagnosis. Thus, it seems that TMD cases may later present OSA, and OSA may develop TMD over time.

The concomitant management of TMD and OSA should follow the usual guidance mentioned above. We must make sure that CPAP is the treatment of choice for moderate-to-severe cases with medical or mood conditions. The use of MAD in mild cases and otherwise healthy moderate cases is an option that may be better accepted and tolerated than CPAP. MAD is also indicated for moderate-and-severe OSA for people who cannot tolerate CPAP. Again, for concurrent TMD/headache and OSA, as described above, physical therapy for oropharyngeal exercise and/or jaw pain can be initiated; CBT may also be proposed [168, 169, 170]. When indicated, a position correcting device may be added for individuals with a dominant supine sleep position; a suggestion that needs further research validation since it is still debated [182, 193]. It is noteworthy to report that MAD are well tolerated in individuals with TMD and OSA, although some tooth and bone changes can occur, as with any type or oral device, these seem to be minimal [194, 195, 196, 197]. A recent study shown that CPAP and MAD are equipotent in individuals with concomitant OSA and sleep bruxism [198, 199].

Headache and OSA are also frequently associated [74, 137, 138]. Headache is a cardinal sign that guides the treating clinician in OSA screening and diagnosis; when pre-existing headaches lessen in frequency or intensity after treatment, it may be an indication that the individual is responding well to CPAP or MAD [200, 201, 202].

The ideal mandibular advancement to reach the most effective AHI reduction is often about 75% of maximal protrusion, although this can be variable between individuals [203, 204]. Not everybody can tolerate such an advanced jaw position and clinicians may gain in finding a good compromise between headache relief and MAD comfort. In a pilot study, we observed equipotent relief of morning headache from a jaw position with upper and lower tooth in an edge-to-edge position *vs.* a 50% maximal protrusion among individuals without OSA or sleep bruxism [205].

The comorbidity between insomnia and sleep apnea (COMISA), is a condition where individuals experience both insomnia and OSA, which may represent a distinct clinical entity with significant health implications [206, 207]. COMISA affects approximately 30–50% of individuals with either condition and often presents with a mismatch between subjective poor sleep quality and objective findings of disrupted sleep; in other words, objective findings do not look abnormal but patients report poor sleep quality and duration [208]. The Sao Paulo epidemiologic sleep study (EPISONO) also calculated the prevalence and incidence of COMISA in the general population in 2007 and 2015 [209]. In 2007 the prevalence of was estimated at 17.64% (95% CI: 15.14 to 20.14). When considering only moderate to severe insomnia and OSA cases, the prevalence of COMISA was reduced to 2.47% (95% CI: 1.45 to 3.49). When stratified per background/original condition, the prevalence of COMISA was 47.01% (95% CI: 43.73 to 50.29) among those with OSA and 39.96% (95% CI: 35.75 to 42.16) among those with insomnia. In 2015, the prevalence of COMISA in the general population increased by 5.31%, reaching 22.95% (95% CI: 19.80 to 26.10) when considering all insomnia and OSA severity levels, but remained stable among moderate and severe cases (2.63%; 95% CI: 1.43 to 3.83). Regarding incidence, this was 16.67% (95% CI: 13.75 to 19.59) in 2015 among individuals without a prior diagnosis in 2007. It decreased to 7.88% (95% CI: 5.77 to 9.99) for those without insomnia or OSA in 2007 but increased to 22.97% (95% CI: 19.67 to 26.26) among those with either condition. A prior diagnosis of OSA increased the risk of developing COMISA by 2.35×, and insomnia increased it by 3.35×, while having either condition raised the risk by 2.91×. Logistic regression confirmed these risks, with odds ratios of 2.68 (*p* = 0.024; 95% CI: 1.141 to 6.328) for OSA and 5.15 (*p* < 0.001; 95% CI: 2.547 to 10.438) for insomnia, adjusted for demographics and health factors. No significant sex differences in COMISA risk were observed. These data suggest a bidirectional interplay between these conditions involving possibly bidirectional mechanisms as well, with OSA contributing to insomnia through frequent arousals and insomnia exacerbating OSA through hyperarousal and sleep instability [206]. COMISA is associated with more severe health consequences than either condition alone, including heightened risks for cardiovascular disease, anxiety, depression and reduced quality of life [210]. Treatment can be challenging, as individuals with COMISA often show lower adherence to OSA therapies like CPAP due to insomnia symptoms, which frequently remain unaddressed. Effective management requires an integrated approach, combining CBT-I with interventions for OSA, such as CPAP or oral appliances, to address both conditions simultaneously [169]. Recognizing and treating COMISA is crucial, as failing to address the overlap may leave patients with persistent sleep issues and heightened health risks [211].

In Switzerland, data from 1236 patients presenting orofacial pain complaints, aged between 10 and 89 years old, and where 69 (1%) were females, was extracted from a national database. It was observed that 142 patients (11.5%) had COMISA, and that characteristics such as BMI, habits such as smoking or alcohol intake, and psychometric measures of anxiety, depression, pain catastrophizing, and stress were generally significantly higher for patients with COMISA compared to patients with isolated conditions [212]. The authors then concluded that orofacial pain patients with COMISA had increased cardiometabolic risk factors compared to those with insomnia or Sleep Disordered Breathing (SDB) alone.

### 5.3 Periodic limb movement during sleep and wake time restless legs syndrome

RLS is a sleep disorder perceived when awake, mostly at end of day and evening; it is characterised by a report of an uncontrollable urge to move and is a sensation that can also be described as painful. RLS is often associated with PLMs during sleep. Both can be present in individuals with CP, as described below. Many individuals with RLS report limb movements during sleep, which can be confirmed by sleep EMG recording of muscle twitches in legs and arms for a diagnosis of PLMs.

RLS is a sensory-motor condition that is frequently observed in individuals with PLMs. RLS is associated with discomfort and pain in legs and arms during wakefulness, characterised by the urge to move. It tends to get worse at night, and is highly prevalent among persons with PLMs, described below. RLS can be primary (idiopathic) or secondary to pregnancy or a variety of systemic disorders, especially iron deficiency and chronic renal insufficiency. The pathophysiology includes dopamine and opiate neurotransmission disturbances, central iron deficiency, and glutaminergic hyperactivity [213, 214]. It tends to run in families and some genetic risk factor candidates have been identified. At this time, many variants have been listed, but no direct therapeutic target has been recognized [215, 216, 217]. RLS is managed with non-pharmacological measures such as walking or mild exercise, massage or baths. Use of prescription medications for RLS includes gabapentin and pregabalin, and dopamine agonists such as pramipexole, ropinirole and rotigotine [218]. Other treatment options include iron-replacement therapy, if reduced body iron stores are identified, or intravenous iron infusion in those who are intolerant to oral iron. Of note, iron deficiency may also be associated with other sleep disturbances including SBD and sleep relationship with Attention-deficit/hyperactivity disorder (ADHD) [219]. If risk of addiction or substance use disorders is thought to be low and the individual is refractory to other treatment strategies, opioids such as tramadol, oxycodone and methadone may be considered as a last resort in the clinician’s tool kit [220, 221]; used with caution in people with OSA or central sleep apnea [222]. Both RLS and PLMs have been associated with some cardiovascular risk. While PLMs seems to be more strongly related to this health risk, the debate is still open [223, 224, 225].

PLM is characterised mainly by brief and recurrent movements of the legs, as well as arms (less common) during sleep. It is prevalent in 4–11% of adults and 5–8% in children and adolescents, increasing with age [226, 227]. Dopaminergic or iron deficiency are associated with such motor manifestations. PLMs are frequent in individuals with RLS and commonly comorbid with OSA, insomnia, narcolepsy or with a rare but critical neurological condition associated with neurodegenerative diseases, REM behaviour disorder. The association of PLMs to the CAP, a physiological biomarker of sleep instability described earlier in this paper, contributes to better delineation of the types of PLMs associated with OSA. For instance, A1 CAP PLMs tend to be reduced under OSA-CPAP treatment *vs.* PLMs in A3 arousal CAP phase [228]. PLMs are diagnosed by PSG recording in a sleep laboratory or by home sleep testing using either EMG or movement sensor to assess the frequency and interval between PLMs events. A diagnosis of PLMs is made in adults if more than 15 events per hour of sleep are scored, while the threshold is 5 events per hours of sleep in children below 18 years (some studies suggest a higher threshold for children under 5 years) [92, 227]. PLMs are most often managed with dopaminergic medications, similar to the approaches for RLS [226, 227, 229]. Clinicians prescribing the above medication for RLS or PLMs have the responsibility to assess if these medications are off label or authorised by their respective governmental drug agency. Special attention must be given in the presence of insomnia and OSA or concomitant mood disorders to achieve best efficacy and effectiveness.

The prevalence of RLS in adults with CP was estimated in a SR to be about 32% [87]. RLS has been reported to be more prevalent among individuals with tension-type headache and migraine than in headache-free controls; 6% *vs.* 3.6% and 16.9% *vs.* 8.7%, respectively [230, 231]. Migraine has been reported to be associated with more severe RLS symptoms, with more difficulty to initiate sleep (*e.g.*, insomnia) and lower sleep efficacy (*e.g.*, non-restorative sleep) [137]. A SR confirmed the importance of the association between migraine and RLS, with RLS prevalence ranging from 15.1% to 62.6% among migraineurs. However, the high unexplained heterogeneity between studies prevented pooling of the data for meta-analysis [232]. Moreover, although PLMs have been associated with sleep bruxism in several reports, not much information is available for PLMs and orofacial pain or headache. While both conditions are associated with disruptions in sleep and may share some overlapping neurophysiological pathways, such as dopaminergic dysfunction or altered sensory processing, specific studies investigating a direct relationship between RLS and orofacial pain are lacking. Further research would be needed to explore potential connections.

## 6. Social determinants of sleep and pain problems

Another important factor for risk and prognosis often overlooked is the presence of social determinants of health, which may play an important role in shaping both sleep and pain experiences, as well as their intricate interactions. Health disparities can be defined as “*inequitable and preventable differences in health outcomes between social groups (e.g., racial and ethnic groups), entrenched in historical, socioeconomic and cultural or political contexts*” [28]. Therefore, factors such as socioeconomic status, education level, employment conditions and access to healthcare may significantly influence sleep quality and pain outcomes. Poor housing conditions, neighborhood noise and limited access to sleep-promoting environments can contribute to insufficient or poor-quality sleep, while financial stress and job insecurity can perpetuate sleep disturbances through heightened arousal and stress [28]. Moreover, food insecurity, psychological distress, everyday discrimination and academic performance should also be accounted [26]. Data from the 2009 Behavioral Risk Factor Surveillance System (n = 323,047 adults) were analyzed to examine insufficient sleep, self-reported as inadequate rest or sleep over a 30-day period. This measure was assessed in relation to various sociodemographic factors (age, sex, race/ethnicity, marital status, region), socioeconomic variables (education, income, employment, insurance status), health behaviors (diet, physical activity, smoking, alcohol use), and indicators of health and functioning (emotional support, BMI, mental and physical health). The findings revealed that insufficient sleep was more prevalent among women, individuals of White or Black/African-American race (*vs.*) those who self-identified as Hispanic/Latino, Asian, Other or Multiracial. In addition, those who were unemployed, uninsured, unmarried, younger, with lower income or educational levels, less physically active and with poorer diets and overall health also present insufficient sleep. Insufficient sleep was also associated with larger household sizes, higher alcohol consumption, and smoking [233].

Social determinants of pain encompass a wide array of factors that operate across multiple levels of organization, profoundly influencing pain experiences, expression, risk, prognosis and impact. Despite widespread acceptance of a biopsychosocial framework, the social dimensions of pain are often underexplored in pain prevention and management contexts [29, 234]. These determinants are dynamic, interacting over time and within socioecological, intersectional, and life course frameworks, creating complex pathways through which social factors influence pain and *vice-versa* [27]. At the interpersonal level, social dynamics such as social comparison, relatedness, support, exclusion, empathy and conflict shape how pain is experienced and expressed. At the community level, the structure of intimacy groups, task groups, social categories and loose associations play a role in modulating pain’s impact and its perception. On a broader scale, societal determinants, including political, economic and cultural systems, alongside their policies and practices, define access to resources, equity in care and societal attitudes toward pain, contributing to disparities and inequities. Interpersonal, community, and societal factors do not operate in isolation but are intricately connected, amplifying or mitigating pain outcomes through multilevel interactions [27].

In addition to shaping pain experiences, these determinants also have reciprocal relationships with pain comorbidities (such as sleep disorders), as chronic pain can reinforce social exclusion, strain relationships and perpetuate economic disadvantages, creating a feedback loop of vulnerability [27]. A critical element in understanding these interactions is the interface between individual intrapersonal processes and the social context, where personal coping mechanisms, emotional resilience and psychological responses mediate social influences. Bridging these insights, a socioecological and multilevel approach highlights opportunities to alleviate the burden and inequities of pain [27]. By incorporating interdisciplinary theories and evidence, addressing social aspects of pain in research and clinical practice could significantly improve prevention and management strategies, ensuring a more equitable and effective response to pain and sleep disorders across populations.

While some socioeconomic and demographic disparities in symptoms have been described specifically for orofacial pain [235, 236, 237], evidence is generally lacking. Likewise, the investigation of social determinants of the association between poor sleep and increased pain is also scarce, yet some studies are available. For example, a study identified social support (inversely associated with sleep disturbance, inflammation and pain severity) in an adult sample with chronic low back pain [238]. Moreover, another study including 820 participants from two predominantly African American socioeconomically disadvantaged neighborhoods in the U.S.A. indicated that psychological distress served as a significant mediator between perceived infrastructure and safety with pain [239]. Although several studies account for basic sociodemographic characteristics as confounders in different analysis concerning this interaction, there remains a significant gap in understanding how broader social determinants—such as access to healthcare, cultural norms and systemic inequities—specifically influence the bidirectional relationship between poor sleep and pain. Addressing this gap is essential for developing targeted interventions that not only mitigate disparities but also improve outcomes for populations disproportionately affected by these interrelated conditions.

## 7. Underexplored or in progress areas of research

There are other yet underexplored or in current research areas that can aid in the understanding of the association between OFP and sleep disorders. One such area is the role of the microbiome and its impact on both sleep and pain related neurotransmitter production. The microbiome plays a pivotal role in maintaining overall health by influencing numerous physiological processes throughout the body [240]. Composed of trillions of microorganisms, including bacteria, viruses, fungi and other microbes, the microbiome is primarily concentrated in the gut but extends to other areas such as the skin, mouth, and respiratory tract. It contributes to digestion and nutrient absorption, synthesizes essential vitamins and regulates the immune system by maintaining a balance between beneficial and potentially harmful microbes. Additionally, the microbiome communicates with various systems, including the endocrine and nervous systems, producing metabolites and signaling molecules that affect metabolism, inflammation and even mood regulation. Disruptions to the microbiome, known as dysbiosis, have been associated with a range of health issues, including gastrointestinal disorders, cardiovascular disease, metabolic syndrome and autoimmune conditions [240]. And lately, these diseases also include sleep disorders [241, 242, 243, 244] and chronic pain conditions [245, 246]. Regarding sleep, for instance, a recent two-sample, bidirectional Mendelian randomization study showed that gut microbiota may be involved in the regulation of sleep, and conversely, changes in sleep-associated traits may also alter the abundance of gut microbiota [247]. OSA has also been associated with dysbiosis, including alterations in fecal microbiome and impaired intestinal barrier function [248], by decreased abundance and diversity in the oropharyngeal area [234], saliva [249] and intestine [250]. Importantly, the latter study reported that this could vary with OSA severity. In addition, a small study showed alterations in Oral–Nasal–Pharyngeal microbiota and in salivary proteins among mouth-breathing children in comparison to nose-breathing children [251]. Regarding chronic pain, probably the most studied condition is fibromyalgia [245, 252]. However, studies have also been conducted in OFP conditions. For instance, a recent review highlighted how in OFP the gut microbiome plays a significant role in pain modulation via the microbiome-gut-brain axis, influencing central pain signaling through direct and indirect pathways [253]. Directly, the vagus nerve connects gut microbiome activity to brain regions like the nucleus tractus solitarius. Indirectly, certain vagal signals from the gut can instigate an anti-inflammatory reflex with afferent signals to the brain activating an efferent response, releasing mediators including acetylcholine that, reduce inflammation [254]. It seems that gut-derived metabolites and molecules modulate pain by crossing the blood-brain barrier and affecting brain structures such as the parabrachial nucleus. Moreover, also recently distinctive salivary oral microbiome have been found in patients with BMS depending on pain intensity compared to healthy subjects [255]. Another two-sample Mendelian randomization analysis assessed the gut microbiota’s effect on TMD, and found that some gut microbiota, including *Coprobacter*, *Ruminococcus torques group*, *Catenibacterium*, *Lachnospiraceae*, *Turicibacter*, *Victivallis*, *MollicutesRF9*, *Methanobacteriales*, *Methanobacteriaceae*, *FamilyXI*, *Methanobacteria* were identified as risk factors, while *Peptococcaceae* provides protection for TMD. Finally, a study showed that Resveratrol, a natural bioactive compound with anti-inflammatory property, alleviated temporomandibular joint (TMJ) inflammatory pain by recovering disturbed gut microbiota (*i.e.*, *Bacteroidetes* and *Lachnospiraceae*) [256]. Moreover, cutting-edge research has shown that the microbiota regulates stress responsiveness in a circadian manner via glucocorticoids, and is necessary to respond adaptively to stressors throughout the day, including sleep/wake transitions and stress-sensitive behaviors [257]. This research area is still in its infancy but has a tremendous potential, offering several opportunities for finding mechanisms and treatments.

The vagus nerve is a key structure with still many unknowns in its association between sleep and pain. Briefly, the vagus nerve is the tenth cranial nerve and a key component of the parasympathetic nervous system, which as well is part of the autonomic nervous system (ANS). The vagus nerve extends from the brainstem to various organs in the body, including the heart, lungs and digestive tract, regulating vital functions such as heart rate, digestion and immune responses. It also plays a role in sensory and motor signaling, linking the brain to the body and facilitating communication between the gut, brain, and other systems. Importantly, the vagus nerve is quite related to the trigeminal nerve, as they are interconnected anatomically at the level of the rostral medulla, and this interaction facilitates cross-talk between somatosensory input from the trigeminal system and autonomic responses mediated by the vagus nerve, influencing processes like pain perception, inflammation and homeostasis [258]. Indeed, a functional magnetic resonance (fMRI) study using non-invasive vagal nerve stimulation (nVNS) showed a complex network involving nVNS including the hypothalamus, the subthalamic nucleus (STN), the pontine nucleus, and the parahippocampal gyrus, which could partially explain the connection between the vagus nerve and the trigeminal autonomic reflex [259, 260]. Likely, this connection is why different studies used or are currently using different methods of vagal nerve stimulation with therapeutic purposes to manage OFP disorders, specially headaches [261]. The influence of the ANS in TMD and other orofacial pain conditions has been demonstrated, for example in the OPPERA study, where it was seen that heart rate at rest and cardiac autonomic function were associated with the incidence and persistence of TMD, respectively [262]. This influence is also obvious in several headache disorders with autonomic features [263]. The connection between the ANS, and sleep disorders such as insomnia, and OSA is well-documented. In insomnia, ANS decompensation can cause hyperarousal, and thus insomnia [264]. In OSA, similarly than in insomnia, there is increased sympathetic activity, reduced parasympathetic activity and less consistently found low heart rate variability [265]. Moreover, OSA therapy selectively improves autonomic function measures [266]. Nonetheless, reports also indicate inducement and aggravation of OSA with vagal nerve stimulation, a reason why the vagus nerve and its stimulation in the presence of OSA should be studied in more detail [267].

Finally, another understudied area is the relationship between awake/sleep position and OFP. For instance, the sometimes observed forward head posture in OSA patients. Older studies using cephalometric measures have observed large cranio-cervical angle than in controls, hypothesizing that hat upper airway obstruction may trigger an increase in the cranio-cervical angulation [268]. Other studies showed that the more severe the OSA, the more severe the head extension [269], and that in comparison to controls, in OSA subjects, the greater the severity of OSA, the greater the head hyperextension and anteriorization [270]. Although one could speculate that this head position could lead to head or neck pain, this relationship is still controversial, with age as a relevant confounding factor [271]. Finally, positional obstructive sleep apnea (POSA), is a well known OSA subtype that exhibits distinct endotypic and phenotypic characteristics [272, 273], and it would be interesting to understand if the association between OSA and pain, including OFP, changes as a function of POSA presence.

## 8. Limitations and other future directions

The main limitation of this review is that it is not systematic in nature, and therefore there is some bias associated with it. Although a thorough search was performed, this review lacks a standardized methodology for selecting, appraising and synthesizing evidence, which can introduce selection bias and reduce reproducibility. Moreover, the absence of systematic inclusion criteria makes it vulnerable to “cherry-picking” studies that support a particular viewpoint while overlooking contradictory evidence. Because this type of reviews typically do not include quantitative synthesis, they cannot provide definitive effect sizes or assess heterogeneity across studies. Nonetheless, we selected the current format to give a broad vision of the interlacement of several different topics in order to have a more clinical outlook.

Moving forward, the future of research and clinical care in the interplay between sleep and orofacial pain requires a multifaceted approach. Longitudinal and population-based studies should focus on elucidating causal pathways between sleep disorders and orofacial pain while incorporating the role of social determinants such as socioeconomic status, access to healthcare and systemic inequities. These future studies must leverage advanced methodologies, including genetic and epigenetic analyses, to deepen our understanding of phenotypes and endotypes that drive individual variability in these conditions.

Personalized medicine must be at the forefront, utilizing tools like artificial intelligence and machine learning to analyze complex datasets, predict treatment responses and design-targeted, patient-specific interventions [274, 275]. Clinical trials should evaluate combined treatment approaches, integrating CBT-I, pharmacological interventions that can target both sleep and pain, and novel therapies such as neuromodulation or regenerative medicine. Research should also expand on the role of restorative naps, sleep extension, and circadian rhythm optimization as adjunctive strategies to alleviate pain and enhance sleep quality.

Moreover, efforts should focus on reducing health disparities by designing interventions tailored to underserved and socioeconomically disadvantaged populations. Public health initiatives must prioritize education and awareness campaigns about the interconnectedness of sleep and pain, aiming to reduce stigma and improve access to care. Interdisciplinary collaboration across dentistry, sleep medicine, neurology and public health is essential to create comprehensive care models that address both the biological and sociocultural dimensions of sleep-pain comorbidities. The integration of these approaches has the potential to significantly improve health outcomes and quality of life for all affected individuals while advancing the field through a holistic and equitable lens.
